# Associations between dairy intake and mortality due to all-cause and cardiovascular disease: the Japan Public Health Center-based prospective study

**DOI:** 10.1007/s00394-023-03116-w

**Published:** 2023-03-21

**Authors:** Sanyu Ge, Ling Zha, Tomotaka Sobue, Tetsuhisa Kitamura, Hiroyasu Iso, Junko Ishihara, Kumiko Kito, Motoki Iwasaki, Manami Inoue, Taiki Yamaji, Shoichiro Tsugane, Norie Sawada

**Affiliations:** 1 Environmental Medicine and Population Science, Department of Social Medicine, Graduate School of Medicine, Osaka University, 2-2 Yamadaoka, Suita, Osaka 565-0871 Japan; 2 Public Health, Department of Social Medicine, Osaka University Graduate School of Medicine, Suita, Japan; 3 Department of Food and Life Science, Azabu University, Sagamihara, Kanagawa 252-5201 Japan; 4 Division of Cohort Research, National Cancer Center Institute for Cancer Control, Chuo-Ku, Tokyo, 104-0045 Japan; 5 Division of Epidemiology, National Cancer Center Institute for Cancer Control, Chuo-Ku, Tokyo, 104-0045 Japan; 6 Division of Prevention, National Cancer Center Institute for Cancer Control, Chuo-Ku, Tokyo, 104-0045 Japan; 7 National Institute of Health and Nutrition, National Institutes of Biomedical Innovation, Health and Nutrition, Tokyo, Japan

**Keywords:** Dairy product, Mortality, Cancer, Cardiovascular disease, JPHC study

## Abstract

**Background:**

Some studies have investigated the relation between dairy products and mortality, but with inconsistent results.

**Objective:**

We examined the association between the consumption of dairy products and the risk of all-cause, cancer-related, and cardiovascular disease (CVD)-related mortality.

**Methods:**

From the Japan Public Health Center-based Prospective (JPHC) study, 43,117 males and 50,193 females with no history of cancer or CVD finished the food frequency questionnaire (FFQ) and were included in the study. Intake of dairy products was assessed using the FFQ and adjusted for total energy by using the residual method. We used multivariate Cox proportional hazard models to calculate hazard ratios (HRs) and 95% confidence intervals (95% CIs) for mortality risk in males and females.

**Results:**

14,211 deaths in males and 9547 deaths in females from all causes were identified during an average follow-up of 19.3 years. For males, total dairy consumption was nonlinearly and significantly associated with lower risk of mortality from all causes [the third quartile, HR = 0.87 (0.83, 0.91), the fourth quartile, HR = 0.89 (0.85, 0.94), *P* for nonlinearity < 0.001] and CVD [the third quartile, HR = 0.77 (0.70, 0.85), the fourth quartile, HR = 0.78 (0.70, 0.86), *P* for nonlinearity < 0.001]. Milk and fermented milk intake were inversely associated with all-cause and CVD-related mortality in males. Cheese consumption was inversely associated with CVD-related mortality among males. There was no association between total dairy intake and mortality risk among females.

**Conclusion:**

For Japanese people, consumption of dairy products was associated with a decreased risk of mortality from all-cause and cardiovascular diseases among males.

**Supplementary Information:**

The online version contains supplementary material available at 10.1007/s00394-023-03116-w.

## Introduction

Although dairy products are abundant in proteins, fats, minerals, and vitamins, and are recommended as an important part of the daily diet, the relation between dairy products and better health outcomes remains controversial [1]. Although there have been several studies on the association between dairy consumption and mortality risk in regions such as Northern Europe [2–4], the Middle East [5], Australia [3], and the United States (US) [6–8], the results have been conflicting. The Golestan Cohort Study suggested a lower risk of all-causes and cardiovascular disease (CVD)-related mortality when dairy intake was higher in Iran [5]. In contrast, dairy intake was a risk factor for all-causes, cancer-related, and CVD-related mortality in Swedish Cohorts [3], the Nurses’ Health Studies, and Health Professionals Follow-up Study [8]. Two meta-analyses reported that overall dairy product consumption was not significantly related with the all-cause mortality risk [9, 10]; however, there was high heterogeneity in the range of dairy consumption across populations [11].

In Japan, milk and dairy product intake is much lower than in Western countries. According to a report on dairy consumption by the Food and Agriculture Organization of the United Nations in 2010, the annual mean per capita milk supply was 46.8 kg in Japan, while in Sweden and the US it was 231.2 kg and 218.3 kg, respectively [12]. However, few cohort studies on dairy intake and mortality have been conducted in Japan [13], and the association between the consumption of dairy products and the risk of mortality in Asian countries has not been sufficiently investigated.

Therefore, we aimed to investigate the associations between the intake of total dairy, milk, cheese, and fermented milk and the risk of all-cause, cancer-related, and CVD-related [heart disease (HD) and cerebrovascular disease] mortality in the Japanese population using a Japan Public Health Center (JPHC)-based prospective study.

## Subjects and methods

### Study population

The JPHC study was a prospective cohort of 140,420 participants from two cohorts. Cohort I was established in 1990 and included five public health centers (Iwate, Akita, Nagano, central Okinawa, and Tokyo). There were 61,595 participants, aged between 40 and 59 years. There were 78,825 participants in Cohort II aged between 40 and 69 years, and these belonged to six public health center regions (Ibaraki, Niigata, Kochi, Nagasaki, Okinawa-Miyako, and Osaka) [14]. Participants were informed of the purpose of the study and asked to complete the survey. The survey was completed at the start of the study, and at 5- and 10-year intervals. We considered the second survey (five-year follow-up survey) as the baseline for this analysis because the food frequency questionnaire (FFQ) with 147 items in the five-year follow-up obtained more extensive information about food consumption as compared to the self-administered FFQ with 44 items, which was the baseline survey. This study was approved by the Institutional Review Board of the National Cancer Center, Japan.

**Fig. 1:**
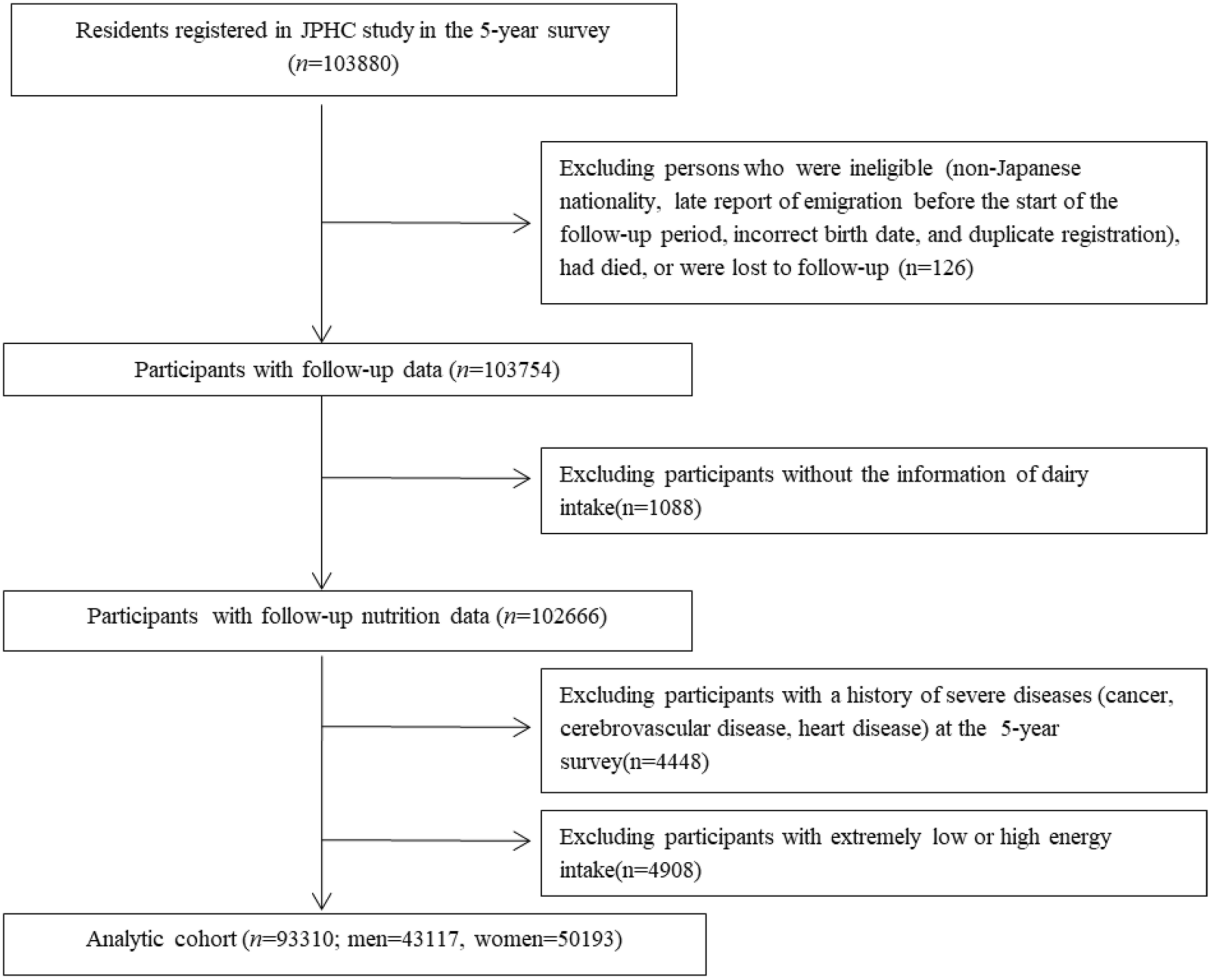
Participant flow chart

A total of 103,880 individuals responded to the second questionnaire. We excluded some ineligible individuals, including individuals who were not Japanese nationals, individuals who reported their emigration late before the start of the follow-up, individuals with incorrect birth dates, duplicate registrations and individuals who died or were lost to follow-up before start of the follow-up (*n* = 126). Among the respondents to the five-year survey, there were 103,754 eligible subjects. Participants with a history of cancer, cerebrovascular disease, or heart disease before the baseline of this study were excluded (*n* = 4,474). We excluded 1062 participants who did not complete the FFQ. We also excluded 4908 participants with extreme energy intake (below 2.5% or over 97.5%). Finally, we included 93,310 participants (43,117 males and 50,193 females) in the final analysis (Fig. 1).

### Follow-up

Participants were followed from the date of the 5-year follow-up survey (second survey) until the date of death, emigration from Japan, or the end of the study, whichever occurred first. Follow-up ended on December 31, 2018, in Iwate, Akita, Nagano, Okinawa Chubu, Ibaraki, Niigata, Kochi, Nagasaki, and Okinawa-Miyako; and on December 31, 2009, in Tokyo; and on December 31, 2012, in Osaka. We followed up participants who died or migrated to other areas through the residential registry. Information on the cause of death was ascertained from the death certificate with the permission of the Japanese Ministry of Health, Labor, and Welfare.

We used the 10th edition of the International Classification of Diseases and Related Health Problems (ICD-10) to classify causes of death. In this study, all-cause mortality, death due to cancer (ICD-10 code: C00-C97), CVD (ICD-10 code: I00-I97), HD (ICD-10 code: I20-I52), and cerebrovascular diseases (ICD-10 code: I60-I69) were included. We also included mortality due to subarachnoid hemorrhage (ICD-10 code: I60), intracerebral hemorrhage (ICD-10 code: I61), and cerebral infarction (ICD-10 code: I63) in the sub-analysis.

### Dietary assessment

We obtained information about the diet by self-administered FFQ. The FFQ investigated the dietary intake of 147 food and beverage items in a 5-year follow-up survey [15]. Questions about the frequency and servings of the following dairy items were asked: milk, cheese, yogurt, Yakult, milk in black tea, and milk in coffee. The frequency of milk, cheese, and yogurt intake was classified into nine categories (never, 1–3 times a month, 1–2 times a week, 3–4 times a week, 5–6 times a week, once a day, 2–3 times a day, 4–6 times a day, and  ≥ 7 times a day). The frequency of Yakult intake was classified into 10 categories (never, 1–3 times a month, 1–2 times a week, 3–4 times a week, 5–6 times a week, once a day, 2–3 times a day, 4–6 times a day, 7–9 times a day, and  ≥ 9 times a day), and the intake of milk in black tea and coffee was classified into five categories (0, 0.5, 1, 2, and  > 3 teaspoons/day). The size of the portion was defined, and the amount was separated into three categories (less than half, the same, and more than 1.5). Total dairy consumption (g/d) and subtype of dairy consumption (g/d) were determined by multiplying the frequency by the relative portion size for each dairy product. Yogurt and Yakult were grouped as fermented group and analyzed together. Because of the comparatively high concentrations of saturated fat and salt in cheese compared to other fermented dairy products, it was considered a distinct subgroup.

Validity was assessed by comparing the FFQ with a 14- or 28-day dietary record (DR), while reproducibility was examined by comparing two five-year follow-up surveys approximately one year apart in a subpopulation [16, 17]. For validity, 565 participants who had completed data for the 28-day DR were included. For reproducibility, we analyzed the data of 498 subjects who had complete data for the both FFQs. Between energy-adjusted dairy consumption from questionnaires and dietary records, Spearman’s correlation coefficients were 0.52 in males and 0.64 in females for Cohort I [17] and 0.69 in males and 0.64 in females for Cohort II [16]. In terms of reproducibility, the respective correlation coefficients for the energy-adjusted intake of dairy products were 0.48 in males and 0.72 in females for Cohort I [18], and 0.69 in males and 0.77 in females for Cohort II [16].

### Statistical analysis

In this study, we analyzed the data separately for males and females. The residual method was used to adjust the dietary intake and dairy products for total energy [19]. Dietary intakes were divided into quartiles. Given that more than 20,000 participants answered that they did not eat cheese or drink fermented milk, the first category (quartile) of cheese and fermented milk intake consisted of participants who did not consume cheese, yogurt, or Yakult. The remaining participants were divided into tertiles. Based on the consumption of dairy products, we used the Cox proportional hazards regression model to determine HRs and 95% CIs for all-cause and cause-specific mortality, using the first category as a reference. Model 1 was adjusted for age at the 5-year survey (continuous) and study area (11 public health centers). Model 2 was further adjusted for smoking status (never, former,  < 20 cigarettes/day,  ≥ 20 cigarettes/day, or missing); frequency of alcohol consumption (never,  < 150 g/week, 150–300 g/week,  > 300 g/week, or missing); body mass index (< 21, 21–22.9, 23–24.9,  ≥ 25 kg/m^2^, or missing); physical activity (quartiles); hypertension with medication (yes, no or missing); self-reported diabetes (yes, no, or missing); green tea (almost never, 1–6 cups/week, 1, 2–4, > 4 cups/d, or missing), coffee (almost never, 1–6 cups/week, 1, 2–4, > 4 cups/d, or missing), energy-adjusted consumption of vegetables (quartiles), fruits (quartiles) and total fat (quartiles); total energy (quartiles); menopausal status (pre- or postmenopausal or missing, only for females); and exogenous hormone use (yes, no, or missing, only for females). In the specific dairy product analysis, Model 3 was further adjusted for consumption of other dairy products. *P* values for linear trend were calculated using the median value in each category as the continuous variable. To analyze the nonlinear association, we calculated *P* values for nonlinearity by including the quadratic terms of the median intake in the regression model [20]. Two-sided *P* values  < 0.05 were considered statistically significant. Analysis was performed using Stata version 16.0 software (StataCorp).

## Results

A total of 93,310 participants with 1,799,379 person-years (average 19.3 years) were followed up in the JPHC study. There were 14,211 males and 9547 females who died due to all causes; 5364 males and 3076 females died due to cancer; 3379 males and 2582 females died due to CVD; 1778 males and 1334 females died due to HD; and 1302 males and 1008 females died due to cerebrovascular disease.

Table 1 shows the characteristics of the study subjects according to total dairy intake. Men who consumed more dairy products drank less alcohol, smoked less, and consumed more fruits and vegetables. Women who consumed more dairy products presented a higher incidence of diabetes and menopause. Both men and women who consumed the most dairy products had the highest fat intake but the lowest total energy intake. Regarding specific dairy intake, total dairy consumption was positively associated with milk and fermented milk consumption in both sexes.

**Table 1:** Baseline characteristics of participants according to quartiles of total dairy products intake

	Men (*n* = 43,117)				*P* value	Women (*n* = 50,193)				*P* value
	First^4^	Second^4^	Third^4^	Highest^4^		First^4^	Second^4^	Third^4^	Highest^4^	
Number	10,780	10,779	10,779	10,779		12,549	12,548	12,548	12,548	
Age,^1^ y	55.5 ± 7.8	54.9 ± 7.6	56.0 ± 7.6	57.1 ± 7.8	< 0.001	56.4 ± 8.0	55.6 ± 7.6	56.2 ± 7.7	56.9 ± 7.9	< 0.001
Body mass index,^1^ kg/m^2^	23.5 ± 3.0	23.6 ± 2.9	23.6 ± 2.8	23.5 ± 2.8	0.005	23.6 ± 3.3	23.5 ± 3.1	23.4 ± 3.1	23.2 ± 3.1	< 0.001
Physical activity,^1^ MET-h/d	37.9 ± 10.4	37.7 ± 10.3	37.6 ± 10.0	36.0 ± 9.8	< 0.001	37.1 ± 9.3	37.5 ± 9.1	37.5 ± 8.9	36.6 ± 8.9	< 0.001
Current smoker,^2^%	52.8	48.5	42.0	35.1	< 0.001	7.5	5.5	4.3	4.6	< 0.001
Alcohol intake (> 1 d/wk),^2^%	58.2	51.7	50.3	37.4	< 0.001	6.4	5.8	4.4	4.6	< 0.001
Past history of diabetes mellitus (yes),^2^%	4.4	5.1	6.8	8.9	< 0.001	2.7	2.6	3.2	4.0	< 0.001
Hypertension with medication (yes),^2^%	17.8	16.3	18.5	20.0	< 0.001	19.3	18.6	18.7	20.6	< 0.001
Postmenopausal status (yes),^2^%	–	–	–	–	–	69.3	70.8	72.4	74.0	< 0.001
Exogenous hormone use (yes),^2^%	–	–	–	–	–	2.4	2.5	2.5	2.8	< 0.001
Green tea intake (> 1 time/d),^2^%	26.1	30.0	31.7	27.4	< 0.001	32.5	36.7	35.1	30.0	< 0.001
Coffee intake (> 1 time/d),^2^%	28.8	34.7	32.8	32.1	< 0.001	32.4	36.8	36.8	35.4	< 0.001
Dietary intake^3^										
Total energy intake, kcal/d	2035 (1644, 2506)	2149 (1710, 2636)	2255 (1886, 2660)	1894 (1598, 2302)	< 0.001	1719 (1370, 2163)	1962 (1561, 2431)	1842 (1586, 2114)	1588 (1351, 1970)	< 0.001
Total fat intake, g/d	45 (34, 57)	49 (40, 59)	52 (43, 62)	58 (50, 68)	< 0.001	48 (39, 57)	51 (44, 59)	53 (47, 60)	57 (50, 64)	< 0.001
Vegetable intake, g/d	147 (93, 226)	161 (107, 232)	174 (118, 248)	171 (112, 250)	< 0.001	192 (128, 277)	196 (139, 275)	205 (147, 278)	190 (130, 270)	< 0.001
Fruits intake, g/d	98 (41, 182)	135 (71, 220)	150 (85, 236)	157 (84, 251)	< 0.001	184 (101, 300)	200 (125, 301)	208 (135, 307)	192 (116, 292)	< 0.001
Dairy products^3^										
Total dairy intake, g/d	7.8 (0.9, 17.3)	59.5 (42.1, 80.6)	153.9 (127.9, 182.6)	321.3 (257.8, 446.1)	< 0.001	25.5 (7.9, 44.0)	105.1 (84.1, 126.7)	198.8 (172.8, 229.5)	375.4 (310.1, 495.9)	< 0.001
Milk intake, g/d	0.5 (0.0, 6.7)	34.5 (15.1, 57.4)	122.7 (94.0, 154.5)	272.8 (213.3, 389.7)	< 0.001	2.6 (0.3, 18.4)	66.1 (39.7, 91.0)	149.7 (114.5, 185.8)	305.4 (236.4, 421.7)	< 0.001
Cheese intake, g/d	0.0 (0.0, 1.2)	0.7 (0.0, 1.7)	0.8 (0.0, 2.0)	0.7 (0.0, 1.9)	< 0.001	0.0 (0.0, 1.1)	0.8 (0.0, 2.3)	1.1 (0.0, 3.6)	0.9 (0.0, 2.5)	< 0.001
Fermented milk intake, g/d	0.0 (0.0, 0.0)	14.5 (0.0, 27.6)	16.9 (0.0, 45.1)	22.4 (0.0, 72.2)	< 0.001	8.7 (0.0, 20.6)	28.4 (11.9, 56.5)	36.4 (13.8, 78.3)	46.0 (14.4, 116.6)	< 0.001

^1^Values are means ± SDs and *P* values were calculated using ANOVA

^2^Values are percentages and *P* values were calculated with a Chi-square test

^3^Intake was adjusted for energy intake with the residual method. Values are medians (interquartile range: IQR). *P* values were calculated using the Kruskal–Wallis test

^4^Total dairy intake was divided into quartiles

Table 2 shows the multivariable-adjusted HRs and 95% CIs for all-cause or cause-specific mortality by energy-adjusted total dairy intake. In men, compared with the lowest group, the multivariable-adjusted HRs for all-cause mortality were 0.92 (95% CI 0.88, 0.97) for the second; 0.87 (0.83, 0.91) for the third; 0.89 (0.85, 0.94) for the highest category of total dairy intake (*P* for nonlinearity  < 0.001). The maximum risk reduction for all-cause mortality was observed in the third category. Similarly, the total dairy intake was inversely and nonlinearly associated with the risk of CVD-related mortality [HR: 0.77 (0.70, 0.85) in the third quartile; 0.78 (0.70, 0.86) in the fourth quartile; *P* for nonlinearity  < 0.001] and HD-related mortality [HR: 0.79(0.68, 0.90) in the third quartile; 0.83 (0.72, 0.95) in the fourth quartile; *P* for nonlinearity = 0.01] among males. In addition, the group with highest total dairy intake was associated with a 30% lower risk of cerebrovascular disease-related mortality [highest vs lowest: HR = 0.70 (0.59, 0.82); P for trend  < 0.001; *P* for nonlinearity = 0.14] as compared with the lowest group in men. Compared to the lowest group of total dairy intake, a 12% risk reduction of cancer-related mortality was found only in the third group, but not the highest intake among men [HR: 0.88 (0.81, 0.95) in the third quartile; 0.98 (0.91, 1.06) in the fourth quartile; *P* for trend = 0.92; *P* for nonlinearity = 0.01]. However, the associations were not significant for risk of all-cause and cause-specific mortality in women.

**Table 2:** Multivariable-adjusted HRs and 95% CIs for all-cause or cause-specific mortality in Japanese adults by energy-adjusted total dairy intake

	Men						Women					
	First^1^	Second^1^	Third^1^	Highest^1^	*P* for trend	*P* for nonlinearity	First^1^	Second^1^	Third^1^	Highest^1^	*P* for trend	*P* for nonlinearity
Median intake (IQR), g/d	7.6 (0.9, 17.3)	59.5 (42.1, 80.6)	153.9 (127.9, 182.6)	321.3 (257.8, 446.1)			25.5 (7.9, 44.0)	105.1 (84.1, 126.7)	198.8 (172.8, 229.5)	375.4 (310.1, 495.9)		
*All-cause*												
Person year	198166	202908	203564	196560			251386	253061	249896	243840		
Number of cases	3903	3302	3372	3634			2616	2197	2250	2484		
HR1	1.00	0.87 (0.83, 0.91)	0.78 (0.75, 0.82)	0.81 (0.77, 0.85)	< 0.001	< 0.001	1.00	0.91 (0.86, 0.97)	0.90 (0.85, 0.95)	0.96 (0.91, 1.02)	0.45	< 0.001
HR2	1.00	0.92 (0.88, 0.97)	0.87 (0.83, 0.91)	0.89 (0.85, 0.94)	< 0.001	< 0.001	1.00	0.97 (0.92, 1.03)	0.96 (0.91, 1.02)	0.97 (0.92, 1.03)	0.38	0.27
*Cancer*												
Person year	198166	202908	203564	196560			251386	253061	249896	243840		
Number of cases	1467	1250	1267	1380			809	765	735	767		
HR1	1.00	0.87 (0.80, 0.93)	0.79 (0.73, 0.85)	0.84 (0.78, 0.90)	< 0.001	< 0.001	1.00	1.00 (0.90, 1.10)	0.94 (0.85, 1.04)	0.98 (0.88, 1.08)	0.54	0.37
HR2	1.00	0.92 (0.85, 1.00)	0.88 (0.81, 0.95)	0.98 (0.91, 1.06)	0.92	0.01	1.00	1.02 (0.92, 1.13)	0.98 (0.88, 1.09)	1.01 (0.91, 1.12)	0.96	0.81
*Cardiovascular disease*												
Person year	198166	202908	203564	196560			251386	253061	249896	243840		
Number of cases	977	797	780	825			735	561	610	676		
HR1	1.00	0.83 (0.76, 0.92)	0.71 (0.64, 0.78)	0.72 (0.66, 0.79)	< 0.001	< 0.001	1.00	0.83 (0.75, 0.93)	0.86 (0.77, 0.95)	0.91 (0.82, 1.01)	0.33	< 0.001
HR2	1.00	0.89 (0.81, 0.98)	0.77 (0.70, 0.85)	0.78 (0.70, 0.86)	< 0.001	< 0.001	1.00	0.91 (0.81, 1.01)	0.92 (0.83, 1.03)	0.92 (0.82, 1.02)	0.20	0.25
*Heart disease*												
Person year	198166	202908	203564	196560			251386	253061	249896	243840		
Number of cases	500	408	406	464			381	296	305	352		
HR1	1.00	0.83 (0.73, 0.95)	0.72 (0.63, 0.82)	0.79 (0.69, 0.89)	< 0.001	< 0.001	1.00	0.86 (0.74, 1.00)	0.83 (0.72, 0.97)	0.91 (0.79, 1.06)	0.38	0.02
HR2	1.00	0.88 (0.77, 1.01)	0.79 (0.68, 0.90)	0.83 (0.72, 0.95)	0.01	0.01	1.00	0.95 (0.81, 1.11)	0.90 (0.77, 1.05)	0.90 (0.78, 1.05)	0.20	0.43
*Cerebrovascular disease*												
Person year	198166	202908	203564	196560			251386	253061	249896	243840		
Number of cases	392	315	307	288			286	213	244	265		
HR1	1.00	0.82 (0.71, 0.95)	0.69 (0.59, 0.80)	0.63 (0.54, 0.73)	< 0.001	0.01	1.00	0.79 (0.66, 0.95)	0.87 (0.73, 1.03)	0.92 (0.77, 1.08)	0.72	0.04
HR2	1.00	0.89 (0.77, 1.04)	0.76 (0.65, 0.89)	0.70 (0.59, 0.82)	< 0.001	0.14	1.00	0.86 (0.72, 1.03)	0.93 (0.78, 1.11)	0.93 (0.78, 1.11)	0.68	0.32

HR1, adjusted for age; study area

HR2, adjusted for age; study area; smoking status; alcohol frequency; body mass index; physical activity; hypertension with medication; self, reported diabetes; green tea; coffee; energy-adjusted consumption of vegetables and fruits; total energy and total fat; menopausal status (only for women), and exogenous hormone use (only for women)

^1^Total dairy intake was divided into quartiles

Table 3 shows the relationship between the intake of milk, cheese, and fermented milk with the all-cause and cause-specific mortality risk. In males, compared to the lowest group of milk intake, the HRs for total mortality were 0.90 (95% CI 0.86, 0.94) for the third and 0.92 (95% CI 0.88,0.97) for the highest group of milk intake (*P* for nonlinearity  < 0.001). The high intake of fermented milk compared with no consumption had a 6% lower risk of all-cause mortality [highest vs lowest: HR = 0.94 (0.90, 0.995); *P* for trend = 0.02] among males. The association between milk intake and cancer-related mortality was not significant but with a nonlinear trend [*P* for trend = 0.45; *P* for nonlinearity = 0.04] in men. Only the high fermented milk intake was inversely associated with cancer-related mortality [highest vs lowest: HR = 0.91 (0.84, 0.99); *P* for trend = 0.04] in males. Milk, cheese, and fermented milk intake were associated with a 19% lower risk [highest vs lowest: HR = 0.81 (0.73, 0.89); *P* for trend  < 0.001; *P* for nonlinearity = 0.03], a 13% lower risk [highest vs lowest: HR = 0.87 (0.78, 0.97); *P* for trend = 0.04], and a 10% lower risk [highest vs lowest: HR = 0.90 (0.81, 0.996); *P* for trend = 0.02] of CVD-related mortality in males, respectively. In males, a lower risk of HD-related mortality was found for milk and fermented milk intake [milk: *P* for trend = 0.04; fermented milk: *P* for trend = 0.01]. An inverse association between milk intake and risk of cerebrovascular disease-related mortality was observed in males [highest vs lowest: HR = 0.74 (0.63, 0.87); *P* for trend  < 0.001]. In women, a 13% risk reduction in CVD-related mortality was found for milk intake in the third group, but not for the highest intake [HR: 0.87 (0.78, 0.98) in the third quartile; HR: 0.93 (0.83, 1.04) in the fourth quartile; *P* for trend = 0.25; *P* for nonlinearity = 0.03]. Fermented milk intake was inversely associated with risk of all-cause mortality among women [highest vs lowest: HR = 0.93 (0.88, 0.99); *P* for trend = 0.15].

**Table 3:** Multivariable-adjusted HRs and 95% CIs for all-cause or cause-specific mortality in Japanese adults by energy-adjusted milk, cheese, and fermented milk intake

Causes of death	Men						Women					
	First^1^	Second^1^	Third^1^	Highest^1^	*P* for trend	*P* for nonlinearity	First^1^	Second^1^	Third^1^	Highest^1^	*P* for trend	*P* for nonlinearity
*All-cause*												
*Milk*												
Median intake (IQR), g/d	0.3 (0, 1.0)	29.1 (16.5, 46.0)	117.2 (91.9, 143.9)	272.8 (214.3, 389.7)			1.2 (0.1, 8.7)	57.5 (38.5, 76.7)	142.9 (118.5, 170.9)	305.4 (224.9, 421.7)		
Person year	196629.6	202325.8	204568.3	197674.2			248941.1	252323.6	251271.6	245645.7		
Number of cases	3912	3269	3335	3695			2676	2141	2209	2521		
HR1	1.00	0.92 (0.88, 0.96)	0.81 (0.78, 0.85)	0.85 (0.81, 0.89)	< 0.001	< 0.001	1.00	0.94 (0.89, 1.00)	0.88 (0.83, 0.93)	0.98 (0.93, 1.03)	0.68	< 0.001
HR2	1.00	0.96 (0.92, 1.01)	0.89 (0.85, 0.94)	0.92 (0.87, 0.96)	< 0.001	< 0.001	1.00	1.00 (0.94, 1.06)	0.94 (0.89, 1.00)	0.99 (0.93, 1.05)	0.51	0.08
HR3	1.00	0.97 (0.92, 1.01)	0.90 (0.86, 0.94)	0.92 (0.88, 0.97)	< 0.001	< 0.001	1.00	1.01 (0.95, 1.07)	0.95 (0.90, 1.01)	1.00 (0.94, 1.06)	0.71	0.16
*Cheese*												
Median intake (IQR), g/d	0	1.0 (0.7, 1.2)	1.6 (1.5, 2.0)	6.1 (4.4, 11.1)			0	1.0 (0.7, 1.2)	1.8 (1.6, 2.5)	7.6 (4.8, 12.4)		
Person year	397166.4	136645.4	134213.7	133172.4			478442	176324	173092	170325		
Number of cases	8131	2026	1984	2070			5772	1241	1214	1320		
HR1	1.00	0.91 (0.86, 0.95)	0.94 (0.90, 0.99)	0.90 (0.86, 0.94)	< 0.001	0.35	1.00	0.84 (0.79, 0.89)	0.88 (0.83, 0.94)	0.91 (0.85, 0.97)	0.09	0.01
HR2	1.00	0.96 (0.91, 1.01)	1.00 (0.95, 1.05)	0.97 (0.92, 1.02)	0.45	0.71	1.00	0.91 (0.85, 0.97)	0.93 (0.88, 1.00)	0.98 (0.92, 1.04)	0.91	0.15
HR3	1.00	0.97 (0.92, 1.02)	1.01 (0.96, 1.06)	0.99 (0.94, 1.04)	0.79	0.56	1.00	0.92 (0.86, 0.98)	0.94 (0.88, 1.01)	0.99 (0.92, 1.05)	0.68	0.23
*Fermented milk (yogurt and Yakult)*												
Median intake (IQR), g/d	0	12.6 (8.5, 14.3)	24.4 (20.5, 32.5)	76.6 (58.2, 123.5)			0	13.9 (9.3, 18.3)	35.9 (28.4, 45.3)	100.8 (76.4, 138.6)		
Person year	332927.2	157346.8	157374.3	153549.7			214727.9	265564.1	262541.2	255348.9		
Number of cases	6433	2639	2535	2604			2589	2287	2323	2348		
HR1	1.00	0.94 (0.90, 0.98)	0.87 (0.83, 0.91)	0.84 (0.80, 0.88)	< 0.001	< 0.001	1.00	0.87 (0.82, 0.92)	0.88 (0.83, 0.93)	0.85 (0.80, 0.90)	< 0.001	< 0.001
HR2	1.00	1.00 (0.95, 1.04)	0.95 (0.90, 0.99)	0.93 (0.89, 0.98)	< 0.001	0.25	1.00	0.93 (0.87, 0.98)	0.95 (0.89, 1.00)	0.92 (0.87, 0.98)	0.07	0.23
HR3	1.00	1.00 (0.96, 1.05)	0.95 (0.91, 1.005)	0.94 (0.90, 0.995)	0.02	0.45	1.00	0.94 (0.88, 0.99)	0.95 (0.90, 1.02)	0.93 (0.88, 0.99)	0.15	0.39
*Cancer*												
*Milk*												
Median intake (IQR), g/d	0.3 (0, 1.0)	29.1 (16.5, 46.0)	117.2 (91.9, 143.9)	272.8 (214.3, 389.7)			1.2 (0.1, 8.7)	57.5 (38.5, 76.7)	142.9 (118.5, 170.9)	305.4 (224.9, 421.7)		
Person year	196629.6	202325.8	204568.3	197674.2			248941	252324	251272	245646		
Number of cases	1435	1260	1278	1391			803	757	732	784		
HR1	1.00	0.95 (0.88, 1.02)	0.85 (0.79, 0.91)	0.89 (0.82, 0.95)	< 0.001	< 0.001	1.00	1.04 (0.94, 1.15)	0.95 (0.86, 1.05)	1.02 (0.92, 1.12)	0.97	0.32
HR2	1.00	0.98 (0.91, 1.06)	0.93 (0.86, 1.01)	1.02 (0.94, 1.10)	0.56	0.02	1.00	1.06 (0.96, 1.18)	0.99 (0.89, 1.10)	1.04 (0.94, 1.16)	0.68	0.76
HR3	1.00	0.99 (0.91, 1.06)	0.94 (0.87, 1.02)	1.03 (0.95, 1.11)	0.45	0.04	1.00	1.07 (0.96, 1.18)	0.99 (0.89, 1.10)	1.05 (0.94, 1.16)	0.65	0.78
*Cheese*												
Median intake (IQR), g/d	0	1.0 (0.7, 1.2)	1.6 (1.5, 2.0)	6.1 (4.4, 11.1)			0	1.0 (0.7, 1.2)	1.8 (1.6, 2.5)	7.6 (4.8, 12.4)		
Person year	397166.4	136645.4	134213.7	133172.4			478442	176324	173092	170325		
Number of cases	2921	805	789	849			1662	452	468	494		
HR1	1.00	0.97 (0.89, 1.05)	1.00 (0.92, 1.08)	1.00 (0.92, 1.08)	0.88	0.89	1.00	0.93 (0.83, 1.03)	1.01 (0.91, 1.13)	1.05 (0.94, 1.16)	0.22	0.62
HR2	1.00	0.98 (0.90, 1.06)	1.03 (0.95, 1.12)	1.05 (0.96, 1.13)	0.22	0.45	1.00	0.95 (0.85, 1.06)	1.03 (0.93, 1.15)	1.09 (0.97, 1.21)	0.08	0.52
HR3	1.00	0.99 (0.91, 1.08)	1.05 (0.96, 1.14)	1.07 (0.98, 1.16)	0.11	0.35	1.00	0.96 (0.86, 1.08)	1.04 (0.94, 1.16)	1.10 (0.98, 1.22)	0.06	0.45
*Fermented milk (yogurt and Yakult)*												
Median intake (IQR), g/d	0	12.6 (8.5, 14.3)	24.4 (20.5, 32.5)	76.6 (58.2, 123.5)			0	13.9 (9.3, 18.3)	35.9 (28.4, 45.3)	100.8 (76.4, 138.6)		
Person year	332927.2	157346.8	157374.3	153549.7			214728	265564	262541	255349		
Number of cases	2425	1014	947	978			778	752	770	776		
HR1	1.00	0.95 (0.88, 1.02)	0.86 (0.80, 0.93)	0.84 (0.78, 0.90)	< 0.001	0.02	1.00	0.89 (0.81, 0.99)	0.91 (0.82, 1.01)	0.89 (0.81, 0.99)	0.15	0.19
HR2	1.00	0.98 (0.91, 1.06)	0.92 (0.85, 0.99)	0.92 (0.85, 0.997)	0.04	0.16	1.00	0.92 (0.83, 1.02)	0.94 (0.85, 1.05)	0.93 (0.84, 1.04)	0.47	0.41
HR3	1.00	0.98 (0.91, 1.06)	0.92 (0.85, 0.99)	0.91 (0.84, 0.99)	0.04	0.16	1.00	0.91 (0.82, 1.02)	0.93 (0.84, 1.03)	0.92 (0.82, 1.02)	0.33	0.34
*Cardiovascular disease*												
*Milk*												
Median intake (IQR), g/d	0.3 (0, 1.0)	29.1 (16.5, 46.0)	117.2 (91.9, 143.9)	272.8 (214.3, 389.7)			1.2 (0.1, 8.7)	57.5 (38.5, 76.7)	142.9 (118.5, 170.9)	305.4 (224.9, 421.7)		
Person year	196629.6	202325.8	204568.3	197674.2			248941	252324	251272	245646		
Number of cases	978	771	789	841			779	537	578	688		
HR1	1.00	0.87 (0.80, 0.96)	0.76 (0.69, 0.83)	0.76 (0.69, 0.83)	< 0.001	< 0.001	1.00	0.83 (0.74, 0.92)	0.79 (0.71, 0.88)	0.91 (0.82, 1.01)	0.27	< 0.001
HR2	1.00	0.91 (0.83, 1.01)	0.81 (0.73, 0.90)	0.80 (0.72, 0.88)	< 0.001	0.01	1.00	0.89 (0.80, 1.00)	0.85 (0.76, 0.95)	0.91 (0.82, 1.01)	0.15	0.01
HR3	1.00	0.93 (0.84, 1.02)	0.83 (0.75, 0.92)	0.81 (0.73, 0.89)	< 0.001	0.03	1.00	0.92 (0.82, 1.03)	0.87 (0.78, 0.98)	0.93 (0.83, 1.04)	0.25	0.03
*Cheese*												
Median intake (IQR), g/d	0	1.0 (0.7, 1.2)	1.6 (1.5, 2.0)	6.1 (4.4, 11.1)			0	1.0 (0.7, 1.2)	1.8 (1.6, 2.5)	7.6 (4.8, 12.4)		
Person year	397166.4	136645.4	134213.7	133172.4			478442	176324	173092	170325		
Number of cases	2044	465	429	441			1660	306	294	322		
HR1	1.00	0.83 (0.75, 0.92)	0.82 (0.74, 0.91)	0.77 (0.69, 0.85)	< 0.001	0.02	1.00	0.75 (0.66, 0.85)	0.78 (0.69, 0.89)	0.81 (0.72, 0.92)	0.02	0.01
HR2	1.00	0.89 (0.80, 0.99)	0.89 (0.80, 0.99)	0.85 (0.76, 0.94)	0.01	0.22	1.00	0.84 (0.74, 0.96)	0.86 (0.75, 0.97)	0.89 (0.78, 1.01)	0.26	0.09
HR3	1.00	0.91 (0.82, 1.01)	0.91 (0.81, 1.01)	0.87 (0.78, 0.97)	0.04	0.30	1.00	0.86 (0.76, 0.98)	0.88 (0.77, 1.00)	0.91 (0.80, 1.04)	0.43	0.16
*Fermented milk (yogurt and Yakult)*												
Median intake (IQR), g/d	0	12.6 (8.5, 14.3)	24.4 (20.5, 32.5)	76.6 (58.2, 123.5)			0	13.9 (9.3, 18.3)	35.9 (28.4, 45.3)	100.8 (76.4, 138.6)		
Person year	332927.2	157346.8	157374.3	153549.7			214728	265564	262541	255349		
Number of case	1567	649	582	581			738	600	617	627		
HR1	1.00	0.95 (0.87, 1.04)	0.82 (0.75, 0.90)	0.77 (0.70, 0.84)	< 0.001	0.03	1.00	0.81 (0.73, 0.90)	0.82 (0.73, 0.91)	0.79 (0.71, 0.88)	< 0.001	0.01
HR2	1.00	1.03 (0.94, 1.13)	0.90 (0.81, 0.99)	0.86 (0.78, 0.95)	< 0.001	0.53	1.00	0.88 (0.78, 0.98)	0.91 (0.81, 1.02)	0.87 (0.78, 0.98)	0.11	0.23
HR3	1.00	1.05 (0.96, 1.16)	0.94 (0.84, 1.04)	0.90 (0.81, 0.996)	0.02	0.99	1.00	0.90 (0.81, 1.01)	0.94 (0.84, 1.05)	0.91 (0.81, 1.02)	0.32	0.49
*Heart disease*												
*Milk*												
Median intake (IQR), g/d	0.3 (0, 1.0)	29.1 (16.5, 46.0)	117.2 (91.9, 143.9)	272.8 (214.3, 389.7)			1.2 (0.1, 8.7)	57.5 (38.5, 76.7)	142.9 (118.5, 170.9)	305.4 (224.9, 421.7)		
Person year	196629.6	202325.8	204568.3	197674.2			248941	252324	251272	245646		
Number of cases	492	410	404	472			417	257	310	350		
HR1	1.00	0.93 (0.81, 1.06)	0.77 (0.67, 0.88)	0.84 (0.74, 0.95)	0.01	< 0.001	1.00	0.76 (0.65, 0.88)	0.80 (0.69, 0.93)	0.86 (0.75, 0.99)	0.23	< 0.001
HR2	1.00	0.97 (0.85, 1.11)	0.83 (0.72, 0.96)	0.86 (0.76, 0.99)	0.02	0.05	1.00	0.83 (0.71, 0.97)	0.87 (0.75, 1.02)	0.85 (0.73, 0.99)	0.10	0.17
HR3	1.00	0.98 (0.85, 1.12)	0.85 (0.74, 0.98)	0.88 (0.77, 1.00)	0.04	0.09	1.00	0.85 (0.73, 1.00)	0.90 (0.77, 1.05)	0.88 (0.75, 1.02)	0.18	0.28
*Cheese*												
Median intake (IQR), g/d	0	1.0 (0.7, 1.2)	1.6 (1.5, 2.0)	6.1 (4.4, 11.1)			0	1.0 (0.7, 1.2)	1.8 (1.6, 2.5)	7.6 (4.8, 12.4)		
Person year	397166.4	136645.4	134213.7	133172.4			478442	176324	173092	170325		
Number of cases	1076	237	233	232			890	148	138	158		
HR1	1.00	0.82 (0.71, 0.94)	0.85 (0.74, 0.98)	0.77 (0.66, 0.89)	< 0.001	0.27	1.00	0.70 (0.59, 0.84)	0.71 (0.59, 0.85)	0.75 (0.63, 0.90)	0.02	0.01
HR2	1.00	0.90 (0.77, 1.04)	0.92 (0.80, 1.07)	0.86 (0.74, 1.00)	0.11	0.59	1.00	0.80 (0.67, 0.96)	0.78 (0.65, 0.94)	0.84 (0.70, 1.01)	0.19	0.05
HR3	1.00	0.91 (0.78, 1.05)	0.93 (0.80, 1.08)	0.89 (0.76, 1.03)	0.23	0.62	1.00	0.82 (0.68, 0.99)	0.80 (0.66, 0.97)	0.85 (0.71, 1.03)	0.26	0.08
*Fermented milk (yogurt and Yakult)*												
Median intake (IQR), g/d	0	12.6 (8.5, 14.3)	24.4 (20.5, 32.5)	76.6 (58.2, 123.5)			0	13.9 (9.3, 18.3)	35.9 (28.4, 45.3)	100.8 (76.4, 138.6)		
Person year	332927.2	157346.8	157374.3	153549.7			214728	265564	262541	255349		
Number of cases	805	373	313	287			393	289	329	323		
HR1	1.00	1.06 (0.94, 1.20)	0.86 (0.75, 0.98)	0.74 (0.65, 0.85)	< 0.001	0.93	1.00	0.75 (0.64, 0.87)	0.83 (0.72, 0.97)	0.78 (0.67, 0.91)	0.04	0.07
HR2	1.00	1.14 (1.01, 1.30)	0.94 (0.82, 1.07)	0.83 (0.72, 0.95)	< 0.001	0.36	1.00	0.81 (0.69, 0.95)	0.93 (0.80, 1.09)	0.87 (0.75, 1.02)	0.42	0.56
HR3	1.00	1.17 (1.03, 1.32)	0.97 (0.84, 1.11)	0.86 (0.74, 0.989)	0.01	0.21	1.00	0.84 (0.72, 0.99)	0.99 (0.84, 1.15)	0.92 (0.79, 1.08)	0.83	0.94
*Cerebrovascular disease*												
*Milk*												
Median intake (IQR), g/d	0.3 (0, 1.0)	29.1 (16.5, 46.0)	117.2 (91.9, 143.9)	272.8 (214.3, 389.7)			1.2 (0.1, 8.7)	57.5 (38.5, 76.7)	142.9 (118.5, 170.9)	305.4 (224.9, 421.7)		
Person year	196629.6	202325.8	204568.3	197674.2			248941	252324	251272	245646		
Number of cases	391	291	321	299			288	225	216	279		
HR1	1.00	0.82 (0.70, 0.95)	0.76 (0.65, 0.88)	0.67 (0.58, 0.78)	< 0.001	0.10	1.00	0.91 (0.76, 1.08)	0.78 (0.65, 0.93)	0.99 (0.84, 1.17)	0.90	< 0.001
HR2	1.00	0.86 (0.74, 1.01)	0.82 (0.70, 0.96)	0.73 (0.62, 0.85)	< 0.001	0.36	1.00	0.98 (0.82, 1.17)	0.84 (0.70, 1.01)	1.01 (0.85, 1.20)	0.98	0.05
HR3	1.00	0.89 (0.76, 1.04)	0.84 (0.72, 0.99)	0.74 (0.63, 0.87)	< 0.001	0.53	1.00	0.99 (0.83, 1.19)	0.85 (0.71, 1.03)	1.02 (0.86, 1.21)	0.92	0.07
*Cheese*												
Median intake (IQR), g/d	0	1.0 (0.7, 1.2)	1.6 (1.5, 2.0)	6.1 (4.4, 11.1)			0	1.0 (0.7, 1.2)	1.8 (1.6, 2.5)	7.6 (4.8, 12.4)		
Person year	397166.4	136645.4	134213.7	133172.4			478442	176324	173092	170325		
Number of cases	786	191	160	165			620	129	130	129		
HR1	1.00	0.87 (0.74, 1.02)	0.79 (0.67, 0.94)	0.75 (0.63, 0.89)	< 0.001	0.06	1.00	0.81 (0.67, 0.99)	0.89 (0.74, 1.08)	0.86 (0.71, 1.04)	0.28	0.59
HR2	1.00	0.93 (0.78, 1.09)	0.87 (0.73, 1.04)	0.82 (0.69, 0.98)	0.04	0.31	1.00	0.90 (0.74, 1.11)	0.97 (0.79, 1.18)	0.94 (0.77, 1.14)	0.68	0.96
HR3	1.00	0.96 (0.81, 1.14)	0.90 (0.76, 1.08)	0.84 (0.71, 1.01)	0.08	0.44	1.00	0.92 (0.75, 1.13)	0.99 (0.81, 1.21)	0.96 (0.78, 1.18)	0.87	0.91
*Fermented milk (yogurt and Yakult)*												
Median intake (IQR), g/d	0	12.6 (8.5, 14.3)	24.4 (20.5, 32.5)	76.6 (58.2, 123.5)			0	13.9 (9.3, 18.3)	35.9 (28.4, 45.3)	100.8 (76.4, 138.6)		
Person year	332927.2	157346.8	157374.3	153549.7			214728	265564	262541	255349		
Number of cases	631	223	217	231			275	246	240	247		
HR1	1.00	0.81 (0.69, 0.94)	0.75 (0.65, 0.88)	0.75 (0.64, 0.87)	< 0.001	< 0.001	1.00	0.87 (0.73, 1.03)	0.82 (0.69, 0.98)	0.79 (0.66, 0.95)	0.03	0.12
HR2	1.00	0.89 (0.76, 1.04)	0.84 (0.72, 0.99)	0.86 (0.73, 1.01)	0.09	0.07	1.00	0.94 (0.79, 1.12)	0.91 (0.76, 1.09)	0.88 (0.73, 1.05)	0.21	0.54
HR3	1.00	0.91 (0.77, 1.06)	0.88 (0.75, 1.04)	0.90 (0.77, 1.06)	0.27	0.16	1.00	0.95 (0.79, 1.13)	0.92 (0.77, 1.14)	0.89 (0.74, 1.08)	0.30	0.63

HR1, adjusted for age; study area

HR2, adjusted for age; study area; smoking status; alcohol frequency; body mass index; physical activity; hypertension with medication; self, reported diabetes; green tea; coffee; energy-adjusted consumption of vegetables and fruits; total energy and total fat; menopausal status (only for women), and exogenous hormone use (only for women)

HR3, furtherly adjusted another dairy intake

^1^Milk intake was divided into quartiles. For cheese and fermented milk, the first group was nonconsumption, and the remaining groups were tertiles

Supplementary Table 1 shows the associations between milk, cheese, and fermented milk intake and the risk of mortality from the stroke subtypes. In males, milk intake was associated with a lower risk of mortality from subarachnoid hemorrhage [highest vs lowest: HR = 0.59 (0.36, 0.98), *P* for trend = 0.05], intracerebral hemorrhage [highest vs lowest: HR = 0.73 (0.56, 0.99), *P* for trend = 0.04] and cerebral infarction [highest vs lowest: HR = 0.71 (0.52, 0.97)]. Cheese intake was inversely associated with mortality due to subarachnoid hemorrhage [highest vs lowest: HR = 0.54 (0.30, 0.97)] in males. Fermented milk intake was not significantly associated with death due to stroke subtypes.

## Discussion

This large prospective cohort study involving 93,310 participants and 23,758 deaths suggested that total dairy intake was nonlinearly associated with a decreased risk of all-cause and CVD-related mortality in males. Regarding specific dairy products, milk intake and fermented milk intake were inversely associated with all-cause and CVD-related mortality risks in males. In addition, higher cheese intake was associated with lower risk of CVD-related mortality among males compared to no intake.

The association between total dairy intake and the risk of all-cause death has been widely researched. A 7% higher risk of total mortality was found comparing the high quintiles group (the mean of dairy intake: 4.2 servings/day) with the lowest group (the mean of dairy intake: 0.8 serving/day) in three cohort studies in the US with high dairy intake, involving 168,153 females and 49,602 males [8]. However, some studies with relatively low dairy intake have suggested different results. The Golestan Cohort found that high intake of total dairy (median intake: 2.4 servings/day in highest group) was a protective factor for all-cause and CVD-related mortality, compared to the lowest group (median intake: 0.4 servings/day in lowest group) [5]. The Prospective Urban Rural Epidemiology (PURE) study included 136,384 participants from 21 countries and found that a high intake of dairy (> 2 servings per day) was associated with a 17% lower risk of total mortality and a 23% lower risk of CVD-related mortality; compared with no intake [21]. However, two meta-analyses found that total dairy intake was not associated with the risk of all-cause mortality [9, 10]. In our study, we found that the high intake of total dairy compared with low intake was inversely and nonlinearly associated with all-cause and CVD-related mortality risk in men with nadirs in the third category, with no enhanced protective effect beyond the third category. However, the dairy intake estimated using the FFQ was not accurate and was overestimated in the subpopulations in the validation study. The medians estimated by DR were 145.0 g/d (97.7, 188.4) in the third and 211.0 g/d (155.3, 279.4) in the highest category; while the corresponding medians estimated from FFQ, respectively, were 222.7 g/d (189.7, 243.1); 378.8 g/d (301.9, 511.8) in men (Supplementary Table 2). Therefore, we speculated that a total daily intake of approximately 145 g (approximately 0.7 serving per day) might be helpful in preventing all-cause and CVD-related mortality in Japanese men. Overall, the range of intake in Japan was much lower than in the US study, but similar to the Golestan Cohort study and the PURE study. The divergent findings across studies might be explained by the different consumption of dairy products between high-intake and low-intake countries, and that dairy products are more likely to be beneficial if the intake is relatively low.

Milk consumption was nonlinearly associated with a lower risk of mortality from all-cause and CVD in males. In another Japanese study, men who consumed milk daily had about a 7% lower all-cause mortality and an 11% lower risk of CVD-related mortality risk compared with men who consumed no milk [13]. An Italian study suggested that milk consumption of up to one serving/day resulted in a 15% decrease in all-cause mortality risk and a 48% decrease in CVD-related mortality risk; however, excessive milk intake was not beneficial [4]. Some previous meta-analyses found differing results; wherein two meta-analyses suggested a null association between milk consumption and risk of mortality due to all-cause or CVD [9, 22]. However, one analysis reported that an increased intake of 200 ml per day could have a 6% lower CVD-related mortality risk [23]. Moreover, one prospective cohort study including 45,339 males and 61,433 females in Sweden suggested that an increased risk of mortality from all causes [highest vs never: females, HR = 1.93 (1.80, 2.06); Males, HR = 1.10 (1.03, 1.17)] and CVD [highest vs never: females, HR = 1.90 (1.69, 2.14); Males, HR = 1.16 (1.06, 1.27)] was found for high milk intake (> 600 g/d) [3]. Three cohort studies in the US found that milk consumption was associated with increased total mortality risk [8]. It is worth emphasizing that the consumption of dairy products and milk in the American and Sweden cohorts was much higher than that in Japan. Milk intake was associated with reduced mortality in countries with a relatively low milk intake [4, 13, 21].

We found that higher cheese intake could reduce CVD-related mortality risks among males, which is consistent with some previous studies [3, 5, 9]. Studies have also reported no association between cheese intake and mortality [2, 4, 8]. In general, cheese intake in Japan was very low, and our reference group did not consume it at all.

Our research suggested that higher intake of fermented milk is inversely associated with risk of mortality from all-cause, cancer, and CVD among males, which is similar to the findings of previous studies [2, 3, 9, 10, 21]. In addition, several meta-analyses have also reported that fermented milk intake might be associated with a decreased risk of cancer and CVD [24, 25].

There are some possible mechanisms explaining our findings. Concerning the mechanism of action, active peptides derived from dairy proteins play an important role in the cardiovascular system. They may exert antihypertensive effects by inhibiting the angiotensin I-converting enzyme [26]. Dairy products are rich in minerals, such as calcium, phosphorus, and potassium, which are related to lowering blood pressure [27–29]. On the other hand, dairy products are high in content of fats, saturated fatty acids, and cholesterol, which have been associated with an increased risk of CVD, especially coronary heart disease in Western countries [30, 31]. This might explain the nonlinear relationship between dairy intake and the risk of mortality from all-cause and CVD. Moreover, a previous study suggested that saturated fatty acids from dietary intake were inversely associated with stroke risk in Japan, which might be due to the low daily intake of saturated fatty acids [32]. Thus, it was thus plausible that total dairy and milk intake were inversely and linearly associated with cerebrovascular disease-related mortality risk. Fermented milk has a greater protective effect on health than regular milk, mainly in terms of better antioxidant activity, anti-hypertension, vitamin enrichment, and probiotics [33–36].

We found no protective association between dairy intake and the risk of cancer-related mortality. Some studies have shown a positive association between dairy intake and cancer mortality [7, 21], while others have reported a negative association [4, 13], and a null association [5, 10]. It is worth noting that dairy products have different effects on different types of cancer. For example, there was strong evidence for dairy intake and an associated decreased risk of colorectal cancer and limited evidence for dairy intake and an associated decreased risk of breast cancer, with an increased risk of prostate cancer [37].

There were also some sex-related differences in our study. The total dairy products and milk were protective against all-cause and CVD-related mortality in males, but not in females. Our results on sex differences were only partly consistent with the results available from previous cohort studies. Milk consumption was associated with a 7% lower risk of all-cause mortality and an 11% lower risk of CVD mortality in males from the Japan Collaborative Cohort Study [13]. However, in some other studies, milk intake had a stronger impact on females’ risk than males. A Netherlands based study reported that a slightly increased risk of all-cause mortality was found for dairy fat intake only in females. A Swedish study reported a stronger association between milk intake and mortality in females than in males, and the adjusted hazard ratio was 1.15 (1.13, 1.17) in females and 1.03 (1.01,1.04) in males. Some studies suggested that women were more likely to take calcium supplements than men and calcium supplements might be inversely related to blood pressure and ischemic heart disease mortality, which might be one reason for sex-related difference [38, 39]. In JPHC study, only 317 (0.6%) women and 98 (0.2%) men reported taking calcium supplements. Given the small sample size, calcium supplementation had little effect on sex differences. In our findings, this difference might be because females have a healthier lifestyle than males. Females have a lower mortality rate, which is related to their lifestyle habits of smoking less, drinking less alcohol, and consuming more vitamins. These factors could distort the actual association of dairy consumption and mortality risk in females. Therefore, more studies on effects of dairy intake on different sexes are needed.

### Limitations and strengths

There were some limitations to this study. First, we only used the dietary information obtained at a 5-year follow-up, which might have resulted in the misclassification of dairy products. Second, several studies have shown that dairy products with different fat content could have different health effects. Low-fat dairy products, especially low-fat milk, were associated with a reduced risk of death, while high-fat dairy products were not. [6, 7]. We were not able to compare the influence of dairy products with different fat content on mortality because of the lack of information included in the questionnaire. Third, the different end dates of follow-up as well as different follow-up durations by area might introduce some bias in the study. However, no material change in the results was observed when we performed the same analysis only in the regions with the same end dates (data not shown). Fourth, the intake of total dairy intake was assessed at a single time point, although participants’ dietary habits could have changed during the follow-up period. Finally, although we adjusted for many confounding factors, there were a few residual confounding factors. This study had some strengths. First, a long-term follow-up (mean: 19.3 years) was conducted for a large number of participants, providing data on a higher number of deaths. Second, there was high reliability (Spearman’s correlation coefficients from 0.52 to 0.69) and reproducibility (coefficients from 0.48 to 0.77) of the dairy-related indicators in the nutrition questionnaire.

## Conclusions

This prospective cohort study showed that total and milk intake were nonlinearly associated with a lower risk of mortality from all-cause and cardiovascular disease in men. Cheese intake was associated with a lower risk of CVD-related mortality in men. Fermented milk was inversely associated with all-cause, cancer- and CVD-related mortality in men. In countries with relatively low dairy consumption, increasing the dairy consumption may have a protective effect on health.

## Supplementary Information

Below is the link to the electronic supplementary material.

Supplementary file 1: (XLSX 19 KB)

## Data Availability

For information on how to gain access to the JPHC data, follow the instructions at https://epi.ncc.go.jp/en/jphc/805/8155.html.

## Abbreviations

CVD: Cardiovascular disease
JPHC: Japan Public Health Center
FFQ: Food frequency questionnaire
HR: Hazard ratio
CI: Confidence interval
HD: Heart disease
ICD-10: International Classification of Diseases and Related Health Problems
DR: Dietary record
PURE: Prospective urban rural epidemiology
